# Novel Formulation of Ionic Liquid-Based Ferrofluids: Investigation of the Magnetic Properties

**DOI:** 10.1021/acs.langmuir.5c00403

**Published:** 2025-05-07

**Authors:** Alessandro Talone, Pierfrancesco Maltoni, Michael Casale, Maryam Abdolrahimi, Sawssen Slimani, Diego Colombara, Luca Leoncino, Patrizia Imperatori, Sara Laureti, Gaspare Varvaro, Davide Peddis

**Affiliations:** † Institute of Structure of Matter (ISM), nM^2^-Lab, National Research Council (CNR), Via Salaria, Km 29.300, Monterotondo Scalo, Rome 00015, Italy; ‡ Department of Science, 19012 University of Rome Tre, via Della Vasca Navale 84, Rome 00146, Italy; § Department of Chemistry and Industrial Chemistry & INSTM RU, 9302 University of Genoa, Via Dodecaneso 31, Genoa 14146, Italy; ∥ Electron Microscopy Facility, Istituto Italiano di Tecnologia (IIT), Via Morego 30, Genova 16163, Italy

## Abstract

This study establishes a viable process to prepare hybrid nanomaterials comprising stable ionic liquid-based ferrofluids (IL-FFs) with tunable magnetic anisotropy and reduced water contamination, where the latter strongly decreases the colloidal stability of the system. Spinel iron oxide magnetic nanoparticles (MNPs) with different compositions (γ-Fe_2_O_3_, Co_0.5_Zn_0.5_Fe_2_O_4_, and CoFe_2_O_4_) and different magnetic anisotropies were synthesized by the polyol method. The particles were coated with dihydrocaffeic acid (DHCA) in tetrahydrofuran (THF) and subsequently transferred directly to 3-ethyl-1-methylimidazolium acetate (EMIMAc), exploring the synergy between intermolecular and covalent bonding to obtain stable dispersions. The evolution of magnetic properties from powder to IL-FFs systems was investigated, allowing us to highlight the synergistic influence of interparticle interaction and magnetic anisotropy on the magnetization dynamics of the nanoparticles.

## Graphical abstract

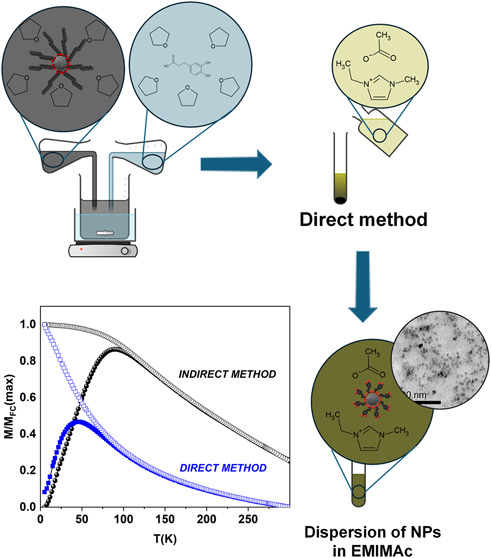

## Introduction

Ionic liquids (ILs) are a class of molten salts characterized by low melting points, often below 100 °C, enabling them to remain liquid near room temperature, in contrast to traditional salts, which are typically solid under these conditions. Comprising solely of ions, ILs boast distinctive chemical structures and properties, setting them apart from both conventional salts and conventional solvents. Their versatility is evident in the ability to fine-tune properties through the selection of different cation–anion pairs, rendering them invaluable across chemistry, engineering, and materials science disciplines.^1,2^ ILs, characterized by their low volatility, nonflammability, and thermal stability,^3,4^ have garnered attention as green solvents across diverse fields, including catalysis,^5,6^ organic synthesis,^7^ and electrochemistry.^8–10^

To enhance the responsiveness of ILs to external stimuli, researchers have explored the incorporation of nanoparticles with different properties,^11–14^ such as single-domain magnetic nanoparticles (MNPs) with superparamagnetic properties at room temperature. The resulting hybrid materials exhibit sensitivity to magnetic fields,^15^ opening interesting perspectives for application in fields such as catalysis, biomedicine, energy (e.g., thermoelectricity), and advanced manufacturing.^16–20^ Ionic liquid-based ferrofluids (IL-FFs) represent innovative avenues in materials science, exhibiting distinctive physicochemical properties, enabling them to respond to an external magnetic field.^21,22^ IL-FFs provide key advantages over water-based systems, including enhanced thermal stability, negligible volatility, and tunable viscosity, making them ideal for high-temperature and electrochemical applications.^23^ Unlike traditional ferrofluids, IL-FFs minimize evaporation and oxidation risks, thus preserving the properties of the ferrofluid over time,^24,25^ but present challenges in colloidal stability due to their higher viscosity and strong interparticle interactions, which can lead to aggregation. Achieving stable dispersions requires tailored surface functionalization strategies to balance dipolar interactions and steric/solvation effects, distinguishing IL-FFs as promising alternatives for specialized applications.^26–29^ In this context, spinel ferrite MNPs offer a versatile platform where the magnetic properties can be finely tailored by manipulating the chemical composition and the distribution of metal cations within their crystalline or amorphous structure.^30–32^

Nevertheless, ILs are hygroscopic, and even small traces of water compromise the dispersion of MNPs therein. Traditional methods involve indirect dispersion routes, requiring controlled atmospheres to prevent water contamination.^9,24^ In such methods, MNPs are directly produced in water and subsequently functionalized, requiring additional steps to incorporate ILs. Freeze-drying procedures are typically undertaken to preserve the dispersions and prevent water contamination-induced instability. In this scenario, efforts to stabilize dispersions of magnetic NPs into ILs have relied on capping agents to enhance stability, leveraging steric and electrostatic repulsions to prevent aggregation.^33–36^

Starting from this complex and fascinating landscape, this study focuses on the development of a formulation with tunable magnetic properties and minimal water contamination (Figure 1). Three MNPs systems with different chemical composition (γ-Fe_2_O_3_, **FO**; Co_0,5_Zn_0.5_Fe_2_O_4_, **CZFO**; CoFe_2_O_4_, **CFO**) have been prepared by the polyol method, ensuring equal morphostructural features and tunable magnetic properties by chemical engineering. The particles have been coated by dihydrocaffeic acid (DHCA) in tetrahydrofuran (THF) and subsequently transferred directly into the applicable IL avoiding direct contact between ILs and water. The IL used as optimal dispersing medium is 3-ethyl-1-methylimidazolium acetate (EMIMAc), an aprotic imidazolium room-temperature ionic liquid, which has a melting point <30 °C. The acetate anion is key to EMIMAc’s strong solvation properties, because of the high hydrogen-bond acceptor ability (making it particularly effective at breaking strong hydrogen bonds in solutes like cellulose or proteins); the imidazolium cation can instead interact with aromatic rings or π-systems, stabilizing aromatic compounds. EMIMAc has been investigated as a reactive solvent for the dissolution of inorganic polymers such as poly­(sulfur nitride) ((SN)*x*),^37^ and can also be used to prepare stable MNPs dispersions owing to the solvation around nanoparticles.^36^ These applications highlight their potential in processing materials that are typically challenging to dissolve.

**Figure 1:**
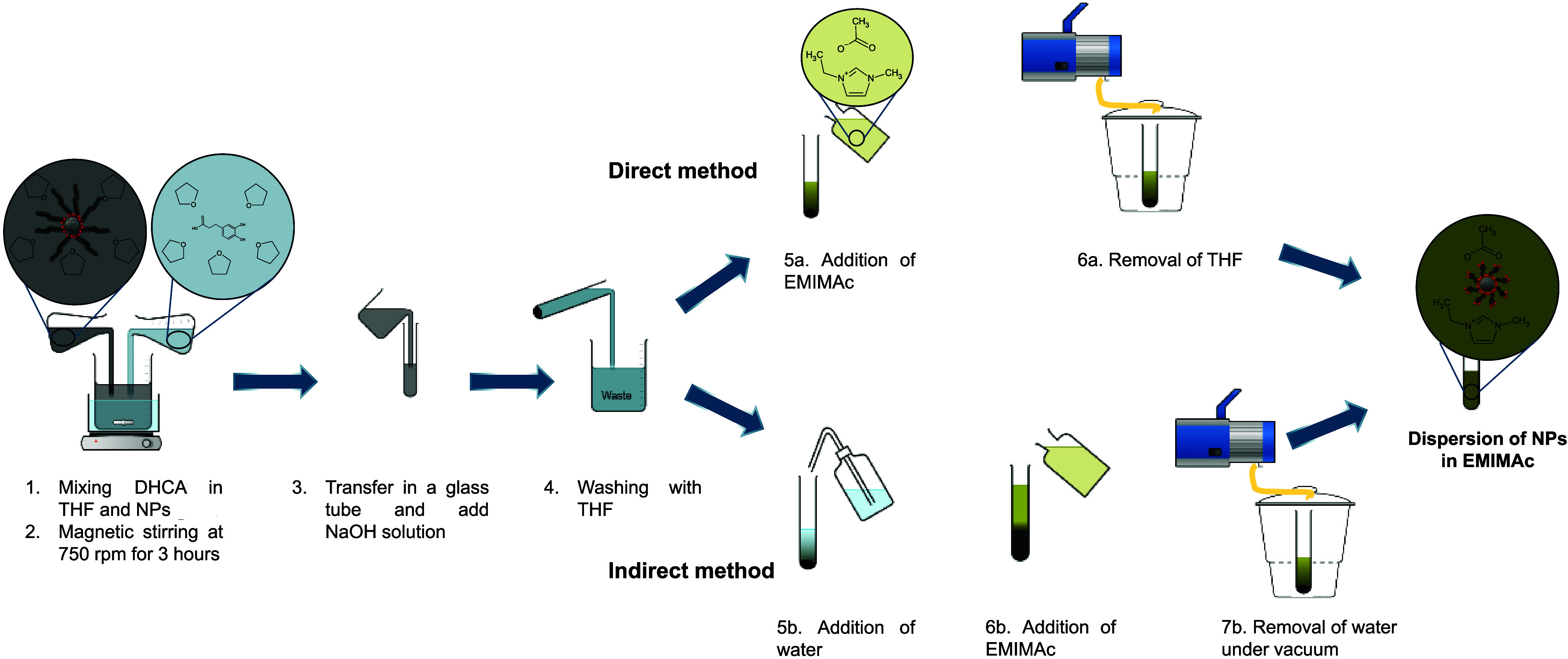
Step-by-step process for dispersing MNPs into ILs after ligand exchange (a) directly without intermediate steps and (b) from a water-based solution.

The preparation of IL-FFs was carried out using both direct (without passing through water) and indirect (passing through aqueous ferrofluids) dispersion methods (Figure 1). Detailed morphological, colloidal, and magnetic comparative studies allow us to investigate the influence of the dispersion methods on the novel properties of IL-FFs. This study highlights that the molecular coating of particles and the magnetic interparticle interactions represent the key physicochemical factors to prepare stable MNPs dispersions in ILs avoiding water content, opening possibilities for various applications in fields such as nanotechnology and materials science. In addition, the evolution of magnetic properties from powder to IL-FFs systems has been investigated in systems with different magnetic anisotropy. This study allows us to discuss the synergistic influence of interparticle interactions and magnetic anisotropy on the magnetization dynamics of the nanoparticles.

## Experimental Section

### Preparation of the Ionic Liquid Ferrofluid (IL-FFs)

Single NPs were synthesized using the polyol method,^38–40^ wherein the polyol serves simultaneously as the solvent, reducing agent, and surfactant. This approach yields a variety of ferrites with adjustable chemical compositions and tunable magnetic properties.^30^ In a typical synthesis, iron­(III) nitrate nonahydrate (Sigma-Aldrich, >98%) and iron­(II) and zinc­(II) nitrate hexahydrate (Sigma-Aldrich, 98%) are dissolved in triethylene glycol (TEG, Sigma-Aldrich, 99%) with the proper ratio, and heated to boiling. After refluxing and magnetic stirring for 3 h, the product is washed and dried. Subsequently, the nanoparticles are sonicated in tetrahydrofuran (THF) and mixed with a water solution of 3,4-dihydroxyhydrocinnamic acid (DHCA, dihydrocaffeic acid; Figure S1a) in alkaline conditions ([NaOH] = 0.5M). The mixture is stirred magnetically in a water bath for several hours, then centrifuged with a metal­(I) hydroxide solution (Sigma-Aldrich) at 3000 rpm, followed by additional THF washes.^41,42^ The supernatant is removed while using a magnet to separate the NPs, and ionic liquid EMIMAc (Solvionic) (Figure S1b) is directly added to achieve stable DHCA-coated MNPs in the IL, yielding the ionic liquid ferrofluid (IL-FF). We refer to this approach as the “direct” method (dm). As a comparison, the dispersions were also prepared with the “indirect” method (im) adding EMIMAc to the aqueous ferrofluid. Both the “direct” and the “indirect” methods involved the prior removal of water by applying a vacuum (Figure 1). Notably, all steps are conducted without a controlled atmosphere. Dispersions of FO, CZFO, and CFO at volume concentrations of ∼1 v/v% are prepared by direct (*X*_dm_ samples) and indirect (*X*_im_ samples) dispersion methods.

### Characterization and Data Elaboration

X-ray powder diffraction (XRPD) is carried out using a Seifert 3003 TT diffractometer equipped with a secondary graphite monochromator, using Cu Kα radiation. The measurements are performed in the 2θ range of 20–80° with a step size of 0.04°, counting 4 s per step.

Transmission electron microscopy (TEM) analysis is carried out by using a JEM-1400Plus microscope equipped with a LaB_6_ thermionic source operating at 120 kV. TEM specimens are prepared by diluting the IL suspended-NPs in mQ water (1:10 vol. ratio), subjecting them to an ultrasonic bath (2 min, 80W), and then drop-casting 2uL onto a commercial TEM support made of a thin carbon film on Cu grid. After mQ water evaporation at ambient conditions, bright-field TEM imaging is performed, and ImageJ software is used for the statistics on particle sizes, intended as Feret’s diameter.^43,44^ Pattern data analysis and electron diffraction simulation have been performed using Scikit-ued, an open-source Python package for data analysis and modeling in electron diffraction.^45,46^

DC magnetic measurements are performed at room and low temperature (*T* = 300 and 5 K, respectively) by using a Quantum Design superconducting quantum interference device (SQUID) that can supply maximum fields of 5 T. Isothermal field-dependent magnetization loops are recorded by sweeping the field in the −5 to +5 T range. Zero field cooled (ZFC) and field cooled (FC) magnetization measurements are carried out by cooling the sample from room temperature to 5 K in zero magnetic field; then, a static magnetic field of 2.5 mT is applied. *M*_ZFC_ is measured during the warmup from 5 to 300 K, whereas *M*_FC_ is recorded during the subsequent cooling. The field dependence of remanent magnetization is measured using the IRM (isothermal remanent magnetization) and DCD (direct current demagnetization) protocols. The DCD curves are measured by applying and removing a progressively higher DC reverse field to a sample previously saturated under a (negative) field of −5 T and by recording, for each step, the value of the remanent magnetization, *M*_DCD_(H), which is then plotted as a function of the reverse field. The IRM curve is obtained starting from a totally demagnetized state by applying a positive magnetic field and measuring the remanence *M*_IRM_(H) when the field is removed; the process is repeated by increasing the field gradually up to 5 T. All of the measurements were corrected by considering the fraction of MNPs.

Dynamic light scattering (DLS) measurements were performed on IL-FFs by a Malvern Zetasizer Nano ZSP equipped with a 10 mW He–Ne red laser (632.8 nm), operating in a backscattered geometry (173°). The sample was irradiated by a laser beam, and the intensity variations of the diffused scattered light areas were measured as a function of time. The intensity variations measured by the detector are generated by the Brownian movement of the particles at the origin of the scattering. At the same temperature and viscosity, the “smaller” particles move more quickly, creating rapid variations in the scattering intensity, while the ’bigger’ particles move more slowly, creating slow intensity variations. This variation is recorded by the autocorrelator, and the particle diffusion coefficient is calculated by the resulting correlation function. The Stokes–Einstein equation then converts the diffusion coefficient into hydrodynamic diameter $d_{H}=\frac{\mathrm{kT}}{3\pi \eta D}$ , where *k* is the Boltzmann constant, *T* is the temperature, η is the viscosity of the medium, and *D* is the diffusion coefficient. Zeta potential (ζ-potential) was also measured to evaluate the stability of the dispersion:^47^ it is the electric potential at the boundary layer around a charged nanoparticle in a liquid medium, thus indicating the degree of electrostatic repulsion between adjacent, similarly charged particles. It is calculated indirectly from electrophoretic mobility by $U/E=\frac{2\varepsilon \zeta F\left(\kappa a\right)}{3\eta }$ , where *U*/*E* is the electrophoretic mobility (m^2^/SV), ζ is the zeta potential (V), ε is the solvent dielectric permittivity (kg m/V^2^s^2^), *F*(κ*a*) is Henry’s function (dimensionless), and η is the viscosity (kg/ms).

## Results and Discussion

Morphological and structural features of the MNPs synthesized via the polyol method were investigated by XRPD and TEM. XRPD patterns of the FO, CZFO, and CFO show reflections corresponding to a cubic phase with spinel structure (Figure S2). No extra phase has been detected. Bright-field transmission electron microscopy (TEM) revealed the presence of MNPs with a diameter of ∼5 nm (see SI, Figure S3a–c), with particle size distributions well described by log-normal functions (Figure S3d).^48–50^ The value of the mean particle size, determined by TEM analysis, is in good agreement with XRD data, confirming the high degree of crystallinity of the materials under investigation (Table 1). This was previously investigated, confirming the MNPs crystallinity^30^ (Figure S4). The field-dependent magnetization loops at 5 K (Figure S5) confirm that the change of composition is effective in tuning the magnetic properties. The samples show a relatively high value of saturation magnetization (*M*_s_) in the range ∼75:90 Am^2^/kg (see Table 2),^51,52^ with small difference among them due to chemical engineering and difference in cationic distribution.^53^ Indeed, TEM analysis shows well-crystallized particles without an amorphous layer; moreover, even for small particles with significant spin canting, M_s_ can exceed the bulk value due to nanoscale changes in the inversion degree.^54^ As expected, the decrease of Co content yields a significant decrease of coercivity and this can be ascribed to the strong single ion anisotropy of Co^2+^ ions;^55^ for CZFO, Zn^2+^ is introduced into the system and preferentially substitutes Co^2+^ in the tetrahedral (A) sites due to its larger ionic radius and low preference for octahedral coordination.^56^ This replacement leads to a redistribution of Fe^3+^ between A and B sites, reducing the occupancy of Co^2+^ in B sites and thereby decreasing the overall anisotropy and coercivity.^53,57^ Reduced remanent magnetization (*M*_R_/*M*_S_) of 0.5 is observed for loops of the CZFO and CFO samples, suggesting the presence of uniaxial anisotropy,^58^ while a significant decrease of *M*_R_/*M*_S_ is observed for the FO sample (∼0.2).^59^ This can be ascribed to the demagnetization field,^60^ or most likely to the presence of a significant fraction of particles still superparamagnetic at 5 K.^61^

**Table 1:** Average Crystallite Size ⟨*D*_XRD_⟩; Average Particle Diameter ⟨*D*_TEM_⟩, Polydispersity (PD), Hydrodynamic Diameter (*d*_H_), and Polydispersity Indices (PDI)^a^

sample	⟨*D*_XRD_⟩ (nm)	⟨*D*_TEM_⟩ (nm)	PD (nm^–1^)	*d*_H_ (nm)	PDI
**FO**	5.5(6)	5.1(5)	9.8(1)	-	-
**FO_dm_**	-	5.5(9)	16.3(1)	42.8 (4)	0.37
**FO_im_**	-	5.7(9)	15.7(1)	40.5 (4)	0.37
**CZFO**	5.2(5)	5.0(1)	2.0(1)	-	-
**CZFO_dm_**	-	5.3(5)	16.9(1)	14.3(2)	0.37
**CZFO_im_**	-	5.1(5)	9.8(1)	18.3(2)	0.37
**CFO**	4.9(5)	4.6(4)	8.6(1)	-	-
**CFO_dm_**	-	4.8(5)	10.4(1)	29.3(3)	0.39
**CFO_im_**	-	5.1(5)	9.8(1)	34.3(3)	0.40

a Uncertainties in the last digit are given in parentheses.

**Table 2:** Average Particle Diameter ⟨*D*_TEM_⟩, Saturation Magnetization (*M*_S_), Reduced Remanent Magnetization (*M*_R_/*M*_S_), and Coercive Field (μ_0_ *H*_C_) Extracted from M­(H) Curves at 5 K; Blocking Temperature (*T*_b_), Maximum Temperature (*T*_max_), and Irreversibility Temperature (*T*_irr_) from ZFC-FC Curves, δ*M* Dip Intensity^a^

sample	⟨*D*_TEM_⟩ (nm)	*M*_S_ (A m^2^kg^–1^)	*M*_R_ / *M*_S_	μ_0_ *H*_C_ (T)	*T*_b_ (K)	*T*_max_ (K)	*T*_irr_ (K)	intensity δ*M*-plot (a.u.)
**FO**	5.1(5)	77(3)	0.25	0.03(1)	38(2)	87(2)	104(3)	–0.54
**FO_dm_**	5.5(9)	67(3)	0.19	0.03(1)	23(2)	45(3)	69(3)	–0.46
**FO_im_**	5.7(9)	64(3)	0.19	0.02(1)	19(1)	45(3)	69(3)	–0.46
**CZFO**	5.0(1)	82(3)	0.48	0.40(1)	94(3)	142(3)	167(6)	–0.21
**CZFO_dm_**	5.3(5)	88(3)	0.50	0.38(1)	68(2)	105(5)	129(4)	–0.08
**CZFO_im_**	5.1(5)	89(3)	0.51	0.38(1)	79(3)	111(5)	138(4)	–0.09
**CFO**	4.6(4)	90(3)	0.56	0.94(1)	160(5)	210(10)	242(10)	–0.16
**CFO_dm_**	4.8(5)	81(3)	0.48	0.90(1)	139(5)	180(5)	216(10)	–0.15
**CFO_im_**	5.1(5)	79(3)	0.56	0.86(1)	132(5)	178(5)	216(10)	–0.15

a Uncertainties in the last digit are given in parentheses.

Then, the nanoparticles were functionalized with dihydrocaffeic acid (DHCA) to enhance their colloidal stability and compatibility with ionic liquids (ILs). The DHCA coating, applied in tetrahydrofuran (THF), provided a protective ligand shell around the nanoparticles, preventing their aggregation and facilitating their transfer into the IL medium.^41^ The preparation of ionic liquid-based ferrofluids (IL-FFs) was carried out using both direct and indirect dispersion methods to achieve stable dispersions of MNPs within the ionic liquid 3-ethyl-1-methylimidazolium acetate (EMIMAc). In the direct method (dm), MNPs coated with DHCA were transferred directly into EMIMAc without the need for intermediate freeze-drying or vacuum processing, thereby minimizing water contamination. Conversely, the indirect method (im) involved first dispersing the nanoparticles in water, followed by a freeze-drying process to remove water, before transferring the dried nanoparticles into the ionic liquid (Figure 1). Bright-field TEM imaging of IL-FFs produced by direct *X*_dm_ (Figure 2a–c) and indirect *X*_im_ methods (Figure 2d–f) allows a direct comparison between the effectiveness of the direct and indirect dispersion methods. For both sets of samples, no significant difference is observed. Particle size distribution shows the same average particle size (Table 1), within experimental error (Figure 2g–i). We also point out that the literature lacks microscopy studies of IL-FF, precisely due to largely unsuccessful protocols of MNPs dispersion into IL.^62,63^

**Figure 2:**
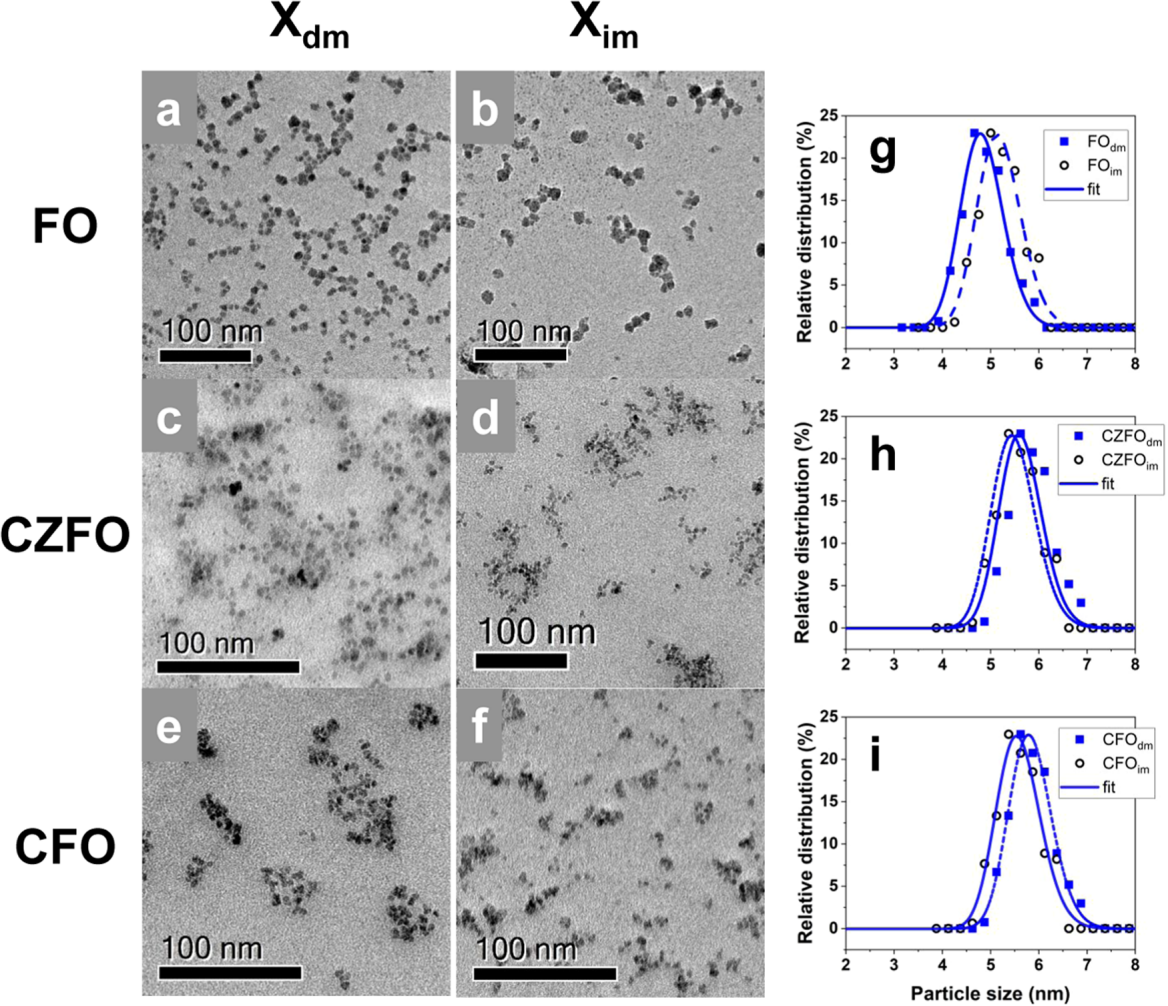
Bright-field TEM images showing: (a, b) Fe_3_O_4_ NPs dispersions obtained by direct and indirect methods (FO_dm_, FO_im_ respectively), (c, d) Co_0,5_Zn_0,5_Fe_2_O_4_ NPs dispersions (CZFO_dm_, CZFO_im_ respectively), and (e, f) CoFe_2_O_4_ NPs dispersions (CFO_dm_, CFO_im_ respectively) and the corresponding size distributions: (g) FO_dm_ (full line), FO_im_ (dashed line), (h) CZFO_dm_ (full line), CZFO_im_ (dashed line), and (i) CFO_dm_ (full line), CFO_im_ (dashed line).

More information about the IL-FFs can be obtained by DLS analysis. First, the ζ-potential is measured for each sample, after a 1:10 dilution. Despite the relatively high concentration (i.e., high darkness), an overall negative potential is measured (>30 mV), indicating that the DHCA-functionalized MNPs dispersed in EMIMAc are negatively charged at the surface and moderately stable owing to electrostatic repulsion. This reflects the colloidal stability provided by the ligand, which is likely due to the interaction with the positive groups of the IL.^64^ The smooth sigmoidal shape of the DLS correlation curves (Figure S6) confirms the moderate colloidal stability, as no secondary steps are present, which may arise from larger aggregates or the tendency of the NPs to precipitate over time^65^ (Figure S7). The particles do not agglomerate or phase separate over the time of measurements (thus after 1 month), and we did not observe any sign of precipitation. The estimated hydrodynamic diameters (*d*_H_) derived from the curves are larger than the values obtained from TEM and XRPD (Table 1). This hints at a larger scattering volume not only due to the presence of the NPs surface ligand and to the additional solvation layers provided by the interacting IL, but also due to clustering of MNPs. Indeed, considering one layer of DHCA equal to ∼1.2 nm, a “monodisperse” system should be of <10 nm. In contrast, d_H_ for FO_dm_, CFO_dm_, and CZFO_dm_ is equal to ∼40 nm, ∼30 nm, and ∼15 nm, respectively (see Table 1). The latter value is presumably due to a larger fraction of coating in the system, as supported by thermogravimetric analysis of the DHCA-coated samples (Figure S8), suggesting a stronger bond between the Zn-doped ferrite and the ligand (∼46% weight loss related to coating for CZFO compared to ∼34 and ∼39% for FO and CFO, respectively), which induces superior colloidal stability. A comparative magnetic investigation of MNPs in the form of powders coated by DHCA and IL-FFs prepared by direct and indirect methods has been carried out. The field-dependent magnetization curves at 5 K (normalized by MNPs mass) show a hysteresis for all of the samples, as a major fraction of the particles is expected to be blocked at low temperature (Figure 3a–c). Since data extracted from hysteresis loops (Table 2) show strong similarities among *X*_dm_ and *X*_im_ samples, we focus on the comparison between powder and dispersion obtained by direct methods that appear as a novelty. The decrease in magnetization observed for FO_dm_ and CFO_dm_ upon coating, in contrast to the nearly unchanged or slightly increased values for CZFO_dm_, can be attributed to the estimation uncertainty of the organic content owing to coating from TG analysis, which is used to normalize the magnetic data. While we cannot exclude a minor effect of the coating on the magnetic properties, the lack of a consistent trend across all samples suggests that this variation is primarily within the experimental error.^34,66^ In order to study magnetization dynamics of the systems, the M vs T was investigated by ZFC-FC protocols (Figure 4a–c). M_ZFC_ curves display a peak at a temperature (*T*_max_), which is directly proportional to the average blocking temperature (*T*_b_).^67^ The proportionality constant (β = 1–2) varies based on the type of T_b_ distribution. An irreversible magnetic behavior emerges below a specific temperature (*T*_irr_), corresponding to the blocking of the largest particles. As the temperature decreases further, the FC curves exhibit a plateau, showing temperature-independent behavior. This indicates the presence of long-range magnetic interparticle interactions, resulting in a magnetically ordered state characterized by high anisotropy.^68^ The temperature-independent behavior is observed in a wider range of temperatures in the series FO, CZFO, and CFO. This can be ascribed to the interplay among interparticle interactions and magnetic anisotropy, increasing the latter with the increase of cobalt content. This scenario is confirmed by the trend of *T*_max_, *T*_irr_, and *T*_b_ convoluted, according to Concas et al.,^69^ that decreases with decreasing cobalt content. It is worth observing that *T*_max_, T_irr_, and T_b_ of powder samples decrease in the corresponding ILs-FF apparently due to the decrease of interparticle distance (i.e., decrease of interparticle interactions). A deeper inspection shows that for FO_dm_ this decrease is ∼40%, whereas for CFO_dm_ and CZFO_dm_, the decrease is smaller (∼30%), although we should expect stronger interactions for CFO and CZFO systems, as their magnetic moment is higher than FO’s. This scenario suggests that the change of behavior from powder to IL-FFs is dominated by a strong interplay between the interparticle interaction and magnetic anisotropy. More information can be obtained by the distribution of magnetic anisotropy obtained by the negative derivative of the thermoremanent magnetization (TRM) curve, as described in the Supporting Information; the TRM was estimated from the difference between *M*_FC_ and *M*_ZFC_,^31^ and it is shown in Figure 5a–c for each sample, with the corresponding *f*(Δ*E*_a_). Moving from powder *X* samples to dispersed *X*_dm_ samples, a shift in the magnetic anisotropy distribution to lower temperatures is observed. It is worth noticing that, despite the same particle volume and similar magnetization, the shift appears less evident for the case of CZFO_dm_ and CFO_dm_, because of the larger anisotropy barrier due to the presence of Co.^70^ This highlights how the particles interact differently depending not solely on the dipolar energy but also on the interplay with magnetic anisotropy. Although the single particle anisotropy is predominant in the Co-doped systems, the longer-range dipolar interactions in the FO systems unveil a different scenario, in terms of interactions.^71,72^ As a further additional evidence that the NP energy barriers regulate the interparticle correlations in Co-doped systems, the irreversible susceptibility component, χ_irr_ = dM_DCD_/dH, was estimated by differentiating the remanence curve of M_DCD_ with respect to the reversal field (Figure 6a–c).^73,74^ In nanoparticle systems, χ_irr_ serves as a measure of the energy barrier distribution linked to the switching field distribution (SFD), the field required to overcome the energy barrier during irreversible magnetization reversal. The SFDs show that the average reversal field (i.e., the field at which the SFD is maximum) is higher for CFO due to the larger cobalt content, reflecting the higher anisotropy (Figure 6c). We can note a slight shift of the maximum of the SFDs for FO_dm_ and CFO_dm_, compared to their powder counterparts, while the SFD for CZFO_dm_ does not significantly change compared to CZFO. This happens to be the system with the smallest hydrodynamic diameter, hinting at the different effect that magnetic interparticle interactions may have on MNPs with different intrinsic magnetic anisotropy.^58^

**Figure 3:**
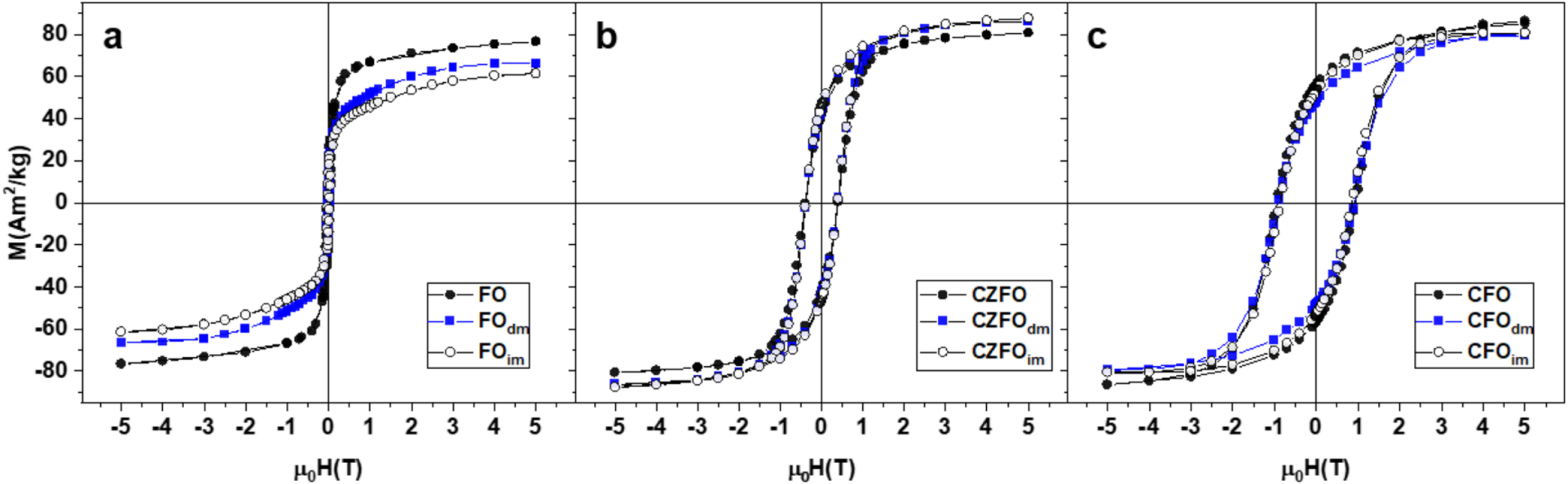
Magnetization (M) vs applied magnetic field (H) recorded at 5 K for (a) powder FO (dots), directly dispersed FO (FO_dm_, empty circles), and indirectly dispersed FO (FO_im_, empty squares); (b) powder CZFO (dots), directly dispersed CZFO (CZFO_dm_, empty circles), and indirectly dispersed CZFO (CZFO_im_, empty squares); and (c) powder CZFO (dots), directly dispersed CFO (CFO_dm_, empty circles), and indirectly dispersed CFO (CFO_im_, empty squares).

**Figure 4:**
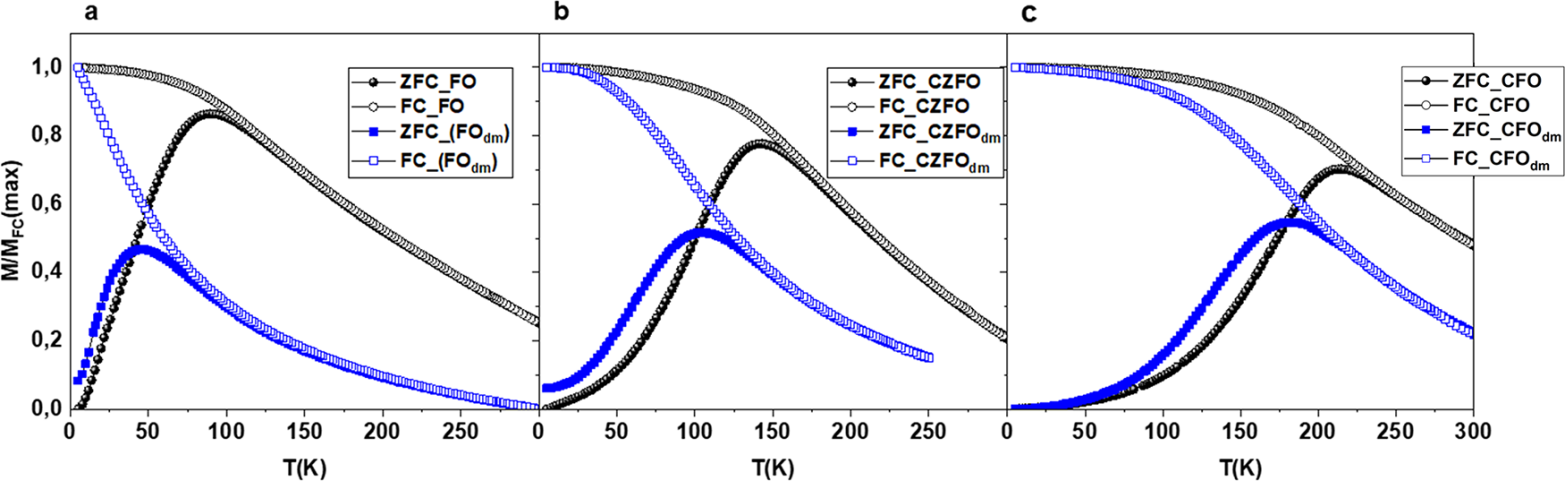
ZFC-FC curves for (a) powder FO (black dots) and FO_dm_ (blue squares); (b) powder CZFO (black dots) and CZFO_dm_ (blue squares); and (c) powder CFO (black dots) and CFO_dm_ (blue squares).

**Figure 5:**
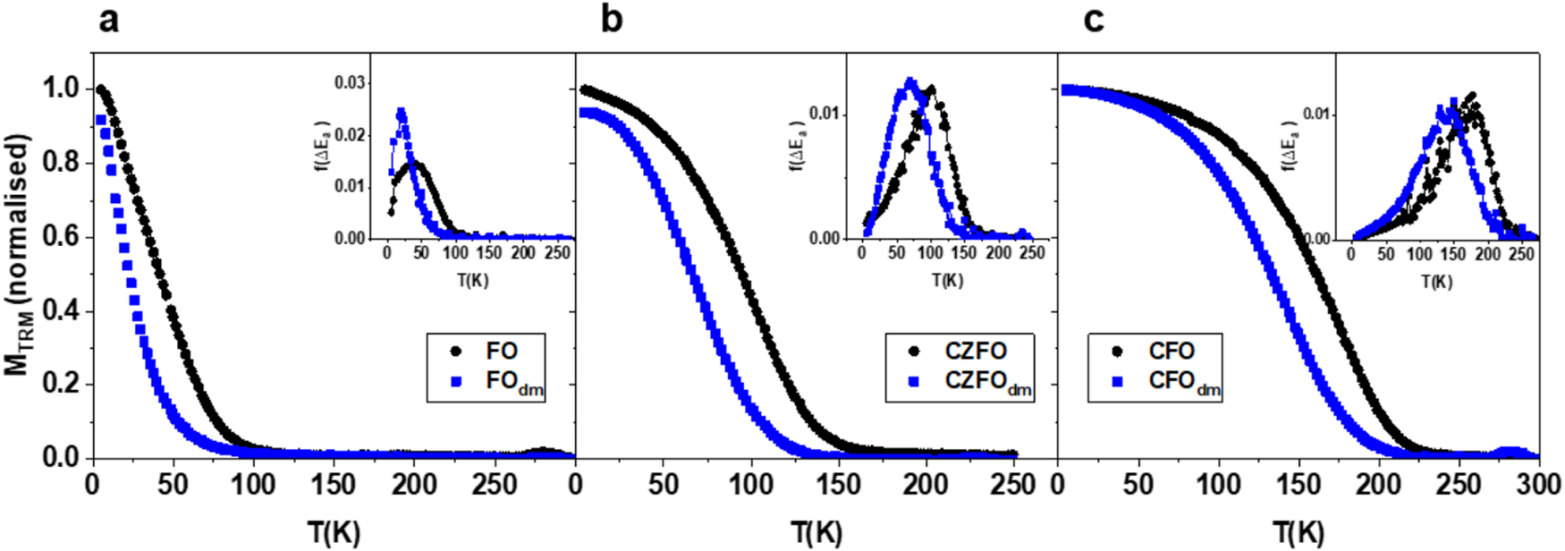
TRM curves for (a) powder FO (black dots) and FO_dm_ (blue squares); (b) powder CZFO (black dots) and CZFO_dm_ (blue squares); and (c) powder CFO (black dots) and CFO_dm_ (blue squares). Corresponding calculated effective magnetic anisotropy energy distribution in the insets.

**Figure 6:**
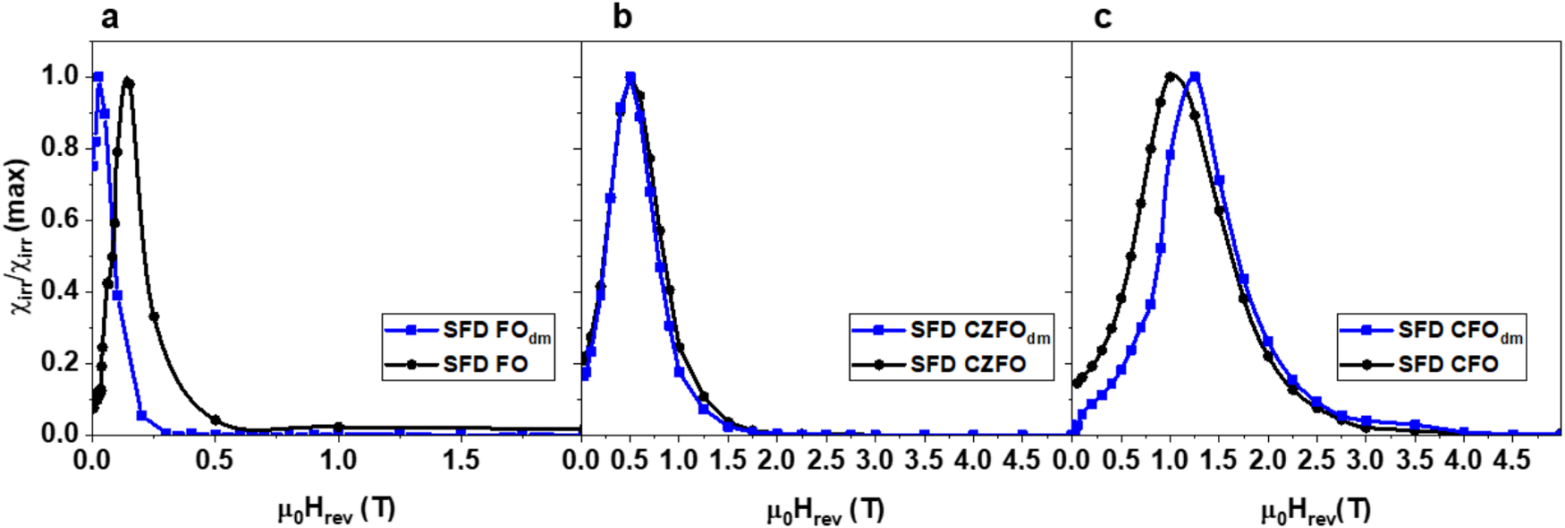
SFD curves for (a) powder FO (black dots) and FO_dm_ (blue squares); (b) powder CZFO (black dots) and CZFO_dm_ (blue squares); and (c) powder CFO (black dots) and CFO_dm_ (blue squares).

A straightforward way to study magnetic interactions in MNPs is to analyze the remanence magnetization plots (see Figure S9 and Supporting Information).^75–77^ If we compare the behavior across the three compositions for particles with the same volume, we expect similar dipolar interactions as their magnetization is only slightly different; actually, the δm dip is deeper for FO (see Figure 7a–c), even though its M_S_ is smaller compared to CZFO and CFO systems, suggesting that the interactions in this system are stronger. Comparing the individual cases, the IL-FFs obtained by the direct method show a change in the δm dip value (empty markers), which decreases with respect to the bare NPs. Such an effect is ascribed to the decrease of dipole–dipole interactions in the dispersion (demagnetizing interactions that typically show a negative dip).^78^ Interestingly, the relative difference for CZFO_dm_ is ∼60%, suggesting that the higher content of ligand (deduced from the largest drop of d_H_ and from TGA, as discussed earlier) is the key factor that increases the stability of particles and suggesting that clustering of MNPs in this case is not significant. On the other hand, the δm dip values of FO_dm_ and CFO_dm_ show a slight decrease of intensity, suggesting that, in these samples, particles are closer in contact, as it could be ascertained from their hydrodynamic diameters, which are larger than that of CZFO_dm_. Furthermore, if we calculate the interaction fields H_in_ (see the SI), those of the powder samples are higher compared to IL-FF (*H*_in_ for FO is 10 mT against 8.9 mT of FO_dm_; for CZFO, it is 32 mT against 12 mT of CZFO_dm_; for CFO, it is 50 mT against 38 mT of CFO_dm_).

**Figure 7:**
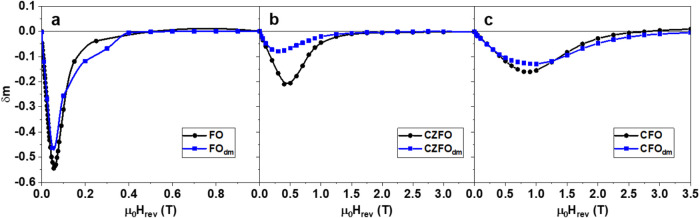
δ*m* parameter calculated from remanent magnetization plots of (a) FO, (b) CZFO, and (c) CFO as powder form (black dots) and as dispersion (blue squares) obtained by the direct method proposed in the study, respectively.

Thus, the magnetic properties of IL-FFs can be finely tuned through precise control over the chemical composition of the nanoparticles. The choice of metal cations and possible substitutions within the spinel ferrite structure significantly influence key magnetic parameters (i.e., magnetization, anisotropy, and magnetization dynamics). These intrinsic modifications directly impact the ferrofluid’s magnetic response, affecting its stability, field-induced structuring, and overall performance in applications requiring tunable magneto-rheological behavior. Our results demonstrate that by tailoring the MNPs’ composition, we can strategically adjust the FFs macroscopic properties, making it a versatile platform for applications in smart materials.

## Conclusions

This study presents a strategy for preparing ionic liquid-based ferrofluids (IL-FFs) by directly dispersing magnetic nanoparticles (MNPs) into 3-ethyl-1-methylimidazolium acetate (EMIMAc), minimizing water contamination and preserving magnetic properties. MNPs with tailored magnetic anisotropy were synthesized via the polyol method and functionalized with dihydrocaffeic acid (DHCA) for enhanced colloidal stability into the IL. The magnetic characterization highlights the critical role of doping in tuning the anisotropy and magnetic moment of the nanoparticles, which directly influence their performance in ionic liquids. The direct dispersion method provides a simplified and efficient pathway for producing IL-FFs while preserving the intrinsic magnetic properties of the MNPs, paving the way for IL-FFs in advanced applications.

## Supplementary Material



## Notes

Ligand and IL molecular formulas; structural characterization of Ferrite NPs; TEM morphological analysis; analysis of DC magnetization measurements; DLS analysis of IL-FFs; TG analysis of DHCA-coated NPs; analysis of remanent magnetization; and references (PDF)
