# Supplementary material for: Novel Formulation of Ionic Liquid-Based Ferrofluids: Investigation of the Magnetic Properties
Source: Langmuir. 2025 May 7;41(19):11977–86. doi: 10.1021/acs.langmuir.5c00403 (PMC12100712; doi:10.1021/acs.langmuir.5c00403)
Supplement: Supplementary file 1 [file la5c00403_si_001.pdf]

Supporting Information

Novel Formulation of Ionic Liquid-based Ferrofluids: Investigation of the Magnetic Properties

Alessandro Talone[1,2], Pierfrancesco Maltoni[1,3], Michael Casale[3], Maryam Abdolrahimi[1,3], Sawssen Slimani[1,3], Diego Colombara[3,4],Luca Leoncino[4], Patrizia Imperatori[1], Sara Laureti[1], Gaspare Varvaro[1], Davide Peddis[1,3]*

**davide.peddis@unige.it*

Number of pages: 9

Number of figures: 9

Ligand and IL molecular formulas........S2
Structural Characterization of Ferrite NPs........S2
TEM Morphological Analysis........S3
Analysis of DC magnetization measurements........S5
DLS analysis of IL-FFs........S6
TG analysis of DHCA-coated NPs........S7
Analysis of remanent magnetization........S7
References........S9

Ligand and IL molecular formulas

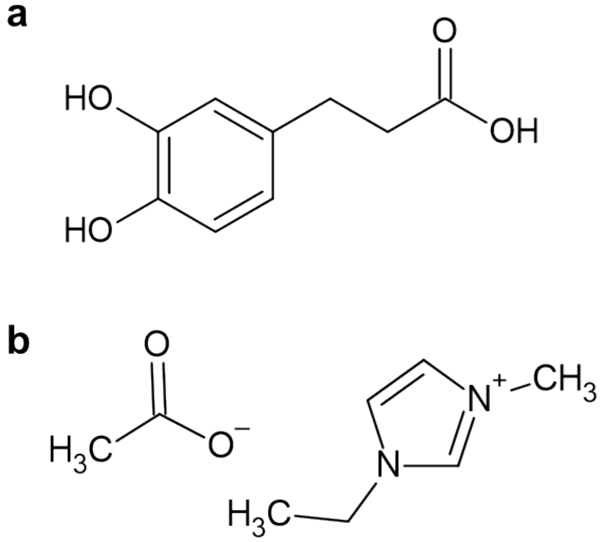

Figure S1: *molecular formulas of (a) 3,4-dihydroxyhydrocinnamic acid (DHCA) (i.e., the capping agent) and (b) 3-ethyl-1-methylimidazolium acetate (EMIMAC) (i.e., ionic liquid).*

Structural Characterization of Ferrite NPs

The average crystallite size was determined by Scherrer equation[1]:

$$\langle D_{XRD} \rangle = \frac{k\lambda}{\beta \cos\theta} \tag{S1}$$

where k is a dimensionless shape factor, λ is the X-ray wavelength, β is the line broadening at half the maximum intensity (also called FWHM) and θ is the Bragg angle. The X-ray powder diffraction (XRPD) patterns (Figure below) show the reflections typical of a cubic spinel structure (PDF card 96_154_0974). No secondary phases were detected.

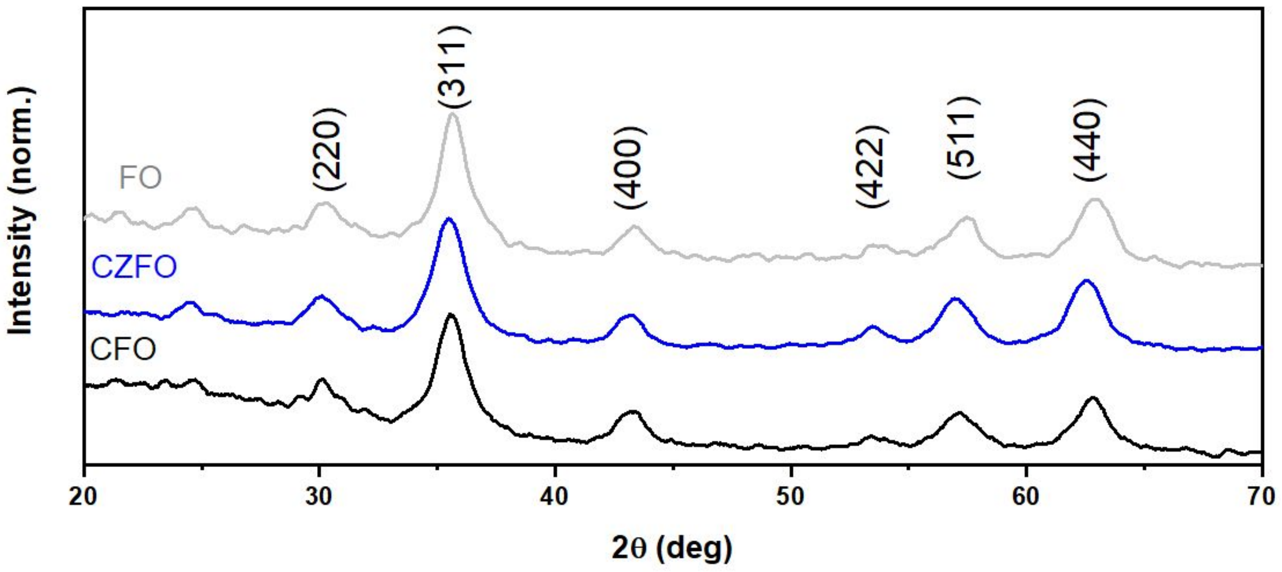

Figure S2: *XRPD patterns of γ-Fe_2O_3 (FO), (b) $Co_{0.5}Zn_{0.5}Fe_2O_4$(CZFO) and (c) $CoFe_2O_4$ (CFO)showing the presence of only cubic spinel phase (PDF. 22-1086).*

TEM Morphological Analysis

Nanoparticles' (NPs) perimeters have been manually drawn and the diameter (D), has been determined according to Ref.[2], as it follows:

$$P = \frac{A}{Dw\sqrt{2\pi}} exp\ - \left[\frac{ln^2\left(\frac{D}{\langle D_{TEM}\rangle}\right)}{2w^2}\right] \qquad \text{(S2)}$$

where *w* represents represents the standard deviation of the logarithms of the diameters, *A* is peak's area and $\langle D_{TEM}\rangle$ the log-normal distribution median. The bright field TEM pictures and corresponding distributions of the powder nanoparticles are shown in the figure below.

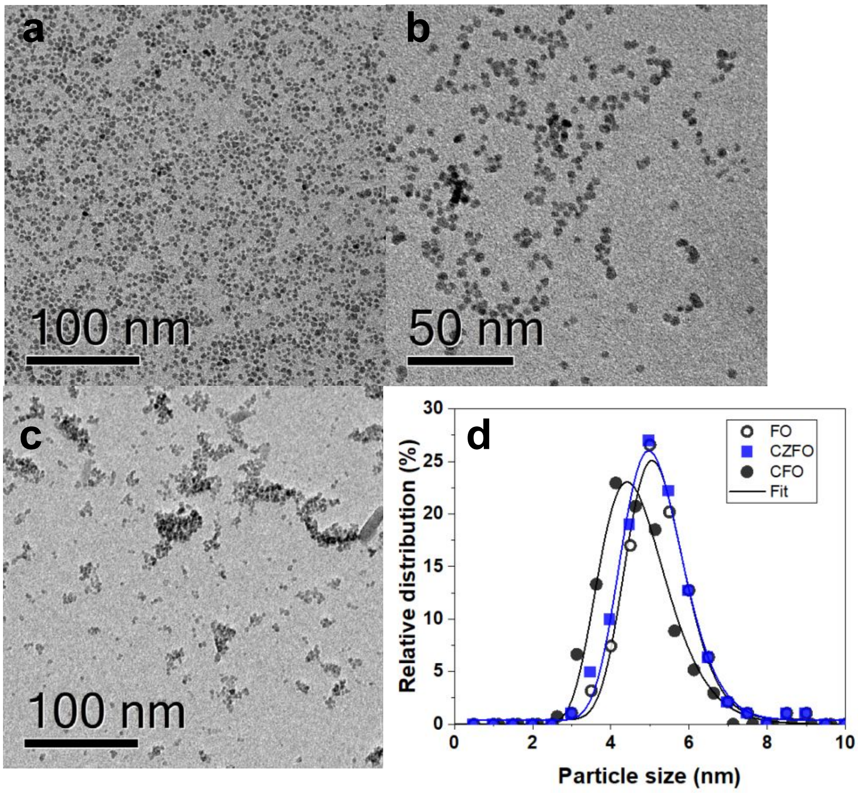

Figure S3: *Bright-field TEM images showing (a) FO, CZFO and (c) CFO NPs post synthesis; the corresponding size distributions are shown in (d).*

The SAED pattern confirms the NPs crystallinity: performing azimuthal integration and applying background subtraction on the diffraction pattern, the obtained SAED profile (blue line) matches to reference pattern obtained from polycrystalline electron diffraction simulation (red histogram) (see Figure below). Pattern data analysis and electron diffraction simulation have been performed using *Scikit-ued*, an open-source Python package for data analysis and modelling in electron diffraction[3,4]. Card #253958 from ICSD database is chosen as reference crystal structure for $Co_{0.5}Zn_{0.5}Fe_2O_4$.

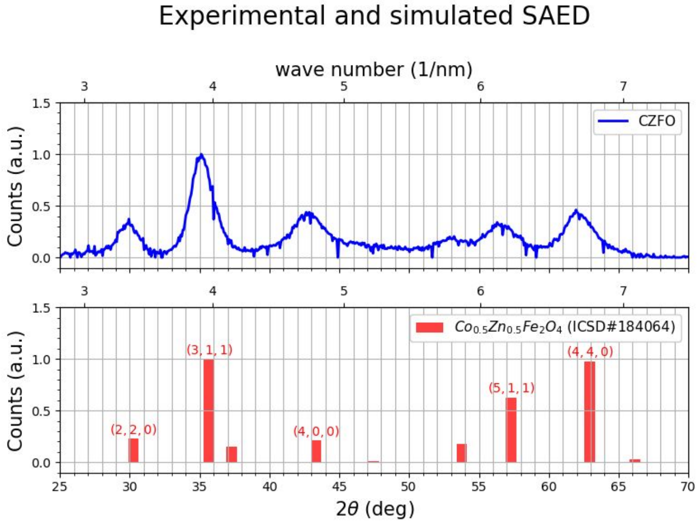

Figure S4: *Experimental and simulated SAED.*

Analysis of DC magnetization measurements

DC magnetization measurements have been carried out using a SQUID magnetometer (Quantum Design) equipped with a superconducting coil ($\mu_0 H_{max}$ = ±5 T). Each sample was placed in the form of powder inside capsules of polycarbonate and was immobilized with epoxy resin, to avoid movements during the measurement (for the IL-FFs, the liquid sample was placed and sealed in the capsule. The dispersion becomes solid-like below ~30°C, therefor it is frozen at low temperatures). The hysteresis loops at 5K for the bare particles are shown in the figure below, for reference.

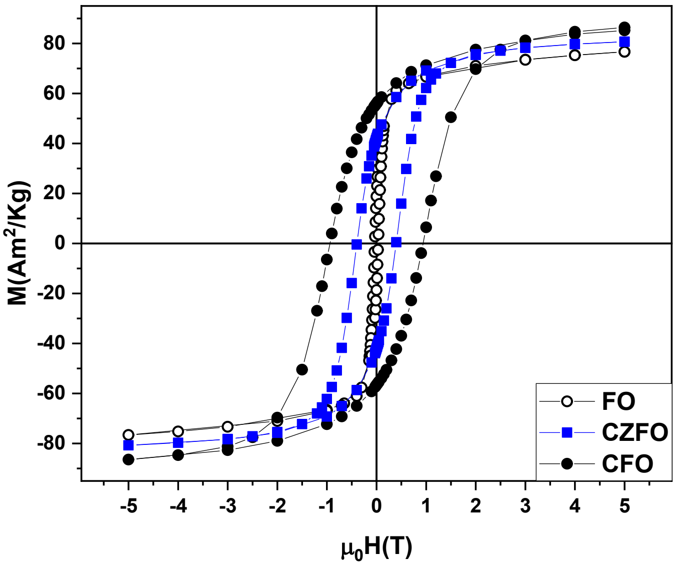

Figure S5: *magnetization (M) vs applied magnetic field (H) recorded at 5 K for (a) powder FO, CZFO and CFO.*

The saturation magnetization M_S was obtained by fitting the hysteresis branch in the high field region[5]:

$$M = M_S\left(1 + \frac{A}{H} - \frac{B}{H^2}\right). \quad \text{(S3)}$$

The parameter A is directly related to the magnetocrystalline anisotropy, as it is proportional to the ratio K_1/Ms^2, where K_1 is the first anisotropy constant[6]. The parameter B, on the other hand, accounts for higher-order corrections and can be influenced by factors such as structural disorder or magnetoelastic effects. In our case, the values of A are comparable among the different samples and follow the expected trend of increasing magnetocrystalline anisotropy from FO to CZFO to CFO.

DLS analysis of IL-FFs

The figure below shows dynamic light scattering (DLS) curves for FO, CFO and CZFO NPs coated with DHCA and dispersed in ILs (IL-FFs). Measurements repeated at regular intervals of time showed values that did not change, demonstrating stable dispersions.

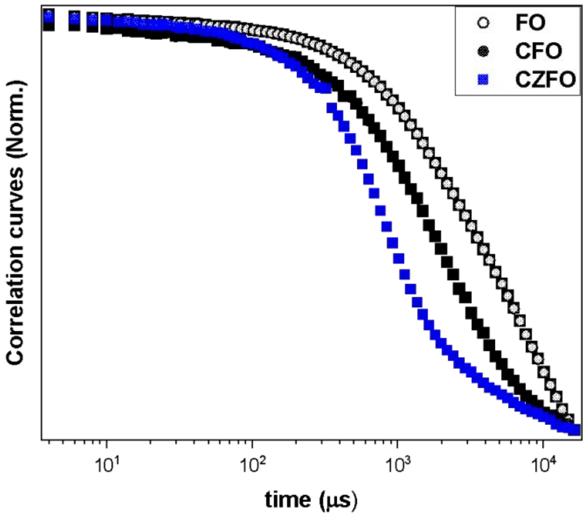

Figure S6: *DLS curves for FO (empty dots), CZFO (blue squares), and CFO (black squares).*

Freshly prepared IL-FFs (we consider for example the FO case) exhibit well-dispersed nanoparticle clusters (~40 nm) with a narrow size distribution and rapid correlation decay, indicating stability for at least one month. After five years of storage, aggregation increases (see **Figure S7**), shifting the size distribution toward larger clusters (>200 nm) while retaining a minor fraction (~40 nm). Despite this, the solution remains stable without precipitation. Mild sonication and redispersion partially restore the smaller fraction, suggesting kinetic trapping rather than irreversible aggregation, though ligand degradation and moisture absorption may limit full recovery. These findings highlight the excellent long-term colloidal stability and reusability of IL-FFs, reinforcing their practical applicability.

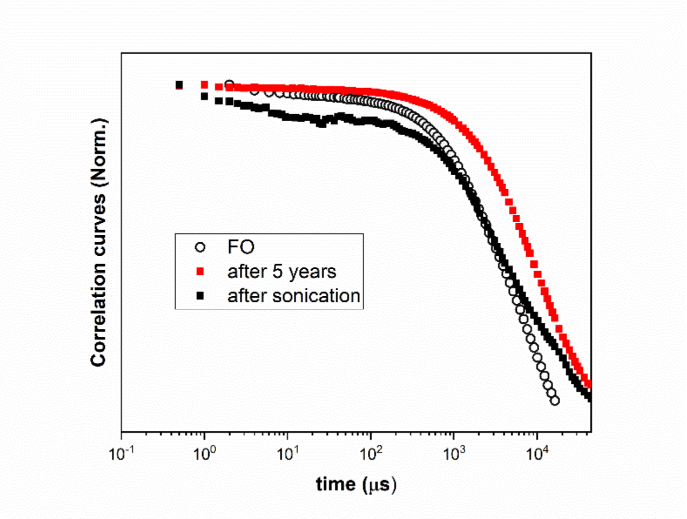

Figure S7: *DLS curves for FO (empty dots), after 5 years (red squares) and after sonication (black squares).*

TG analysis of DHCA-coated NPs

The picture below shows the thermogravimetric curves of the coated samples.

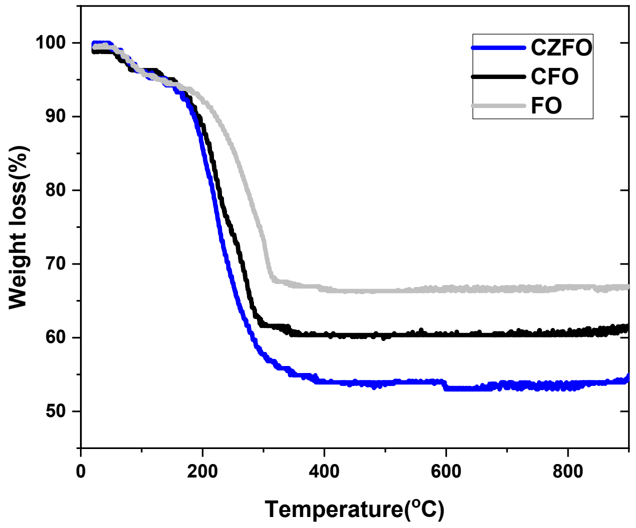

Figure S8: *TG curves for FO (grey), CZFO (blue) and CFO (black).*

Analysis of remanent magnetization

Remanent magnetization was measured by means of Isothermal Remanent Magnetization (IRM) and Direct Current Demagnetization (DCD) protocols. IRM curves are obtained starting from a demagnetized sample at $\mu_0 H = 0$ T and measuring the remanence corresponding to increasing fields $\mu_0 H > 0$; DCD curves are obtained in the same manner, but the measurement starts from $\mu_0 H = 0$ with a sample previously being saturated at $\mu_0 H = -5$ T. This is repeated, increasing the field until saturation in the opposite direction is reached; for reference, IRM and DCD curves of CFO_{dm} sample are reported in the Figure below.

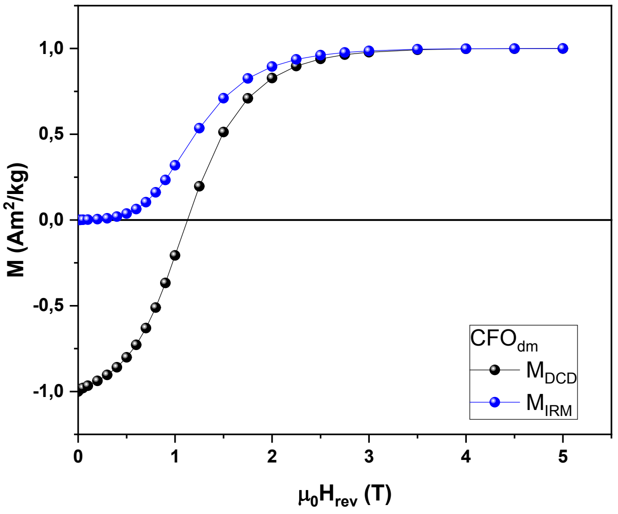

Figure S9: *Remanent magnetization (IRM and DCD) curves for CFO_{dm} at 5K.*

For an ideal assembly of non-interacting single-domain particles with uniaxial anisotropy that reverse their magnetization by coherent rotation, according to the so-called Wohlfarth relation[7]:

$$m_r^{DCD}(H) = 1 - 2m_r^{IRM}(H) \tag{S4}$$

where $m_r^{DCD}(H)$ and $m_r^{IRM}(H)$ denote the reduced terms $M_r^{DCD}(H)/M_r^{DCD}(5T)$ and $M_r^{IRM}(H)/M_r^{IRM}(5T)$, respectively. Any deviation from such behavior can be ascribed to the effect of interactions, which can be quantified in terms of the so-called interaction field:

$$H_{int} = \frac{H_{cr}^{DCD} - H_{cr}^{IRM}}{2} \tag{S5}$$

where H_{cr}^{DCD} and H_{cr}^{IRM} are the remanence coercivity, as the field where M_{DCD} is equal to zero and the field where M_{IRM} achieves its half maximum value, respectively.

Following Wolfarth relation, the two irreversible susceptibilities are related by:

$$\left|\frac{dm_r^{DCD}}{dH}\right| = 2\left|\frac{dm_r^{IRM}}{dH}\right| \tag{S6}$$

with a maximum at the same reverse field. The Wohlfarth's relationship can be rewritten in order to show more clearly the deviation of a real system from the non-interacting case:

$$\delta m(H) = m_r^{DCD} - \left[1 - 2m_r^{IRM}\right] \tag{S7}$$

where the δm parameter is zero in the absence of interactions. Positive deviation from the ideal system is due to interactions promoting the magnetized state (exchange and other positive interactions), whereas negative deviation is due to demagnetizing interactions (e.g. dipole–dipole interactions)[8].

The FC-ZFC curves of a batch of non-interacting particles can provide information on the sample's anisotropy energy distribution, as shown in the following relation[9]:

$$f(\Delta E_a) \propto -\frac{d(M_{FC} - M_{ZFC})}{dT} \tag{S8}$$

It should be underlined that the difference between field cooled (FC) and zero field cooled (ZFC) magnetization can be used instead of measuring the TRM. In fact, for a nanoparticle ensemble it was demonstrated that:

$$M_{TRM}(H,T,t) = M_{ZFC}(H,T,t) - M_{ZFC}(H,T,t) + M_{IRM}(H,T,t) \tag{S9}$$

where M_{IRM} is the isothermal remanent magnetization. Equation 9 indicates that the difference between M_{FC} and M_{ZFC} is a very good approximation of M_{TRM}, being M_{IRM} is negligible in nanoparticle systems.

Once the $d(M_{FC} - M_{ZFC})$ curve is calculated, it can be integrated and normalized, and the temperature at which this final curve reaches the 50% of its maximum value can be assigned to the average sample's T_b. Indeed, at a given temperature T^*, the NPs can be divided into two subpopulations: the blocked particles and the superparamagnetic (SPM) ones. By integration of the areas under the curve, the relative $f(T)$ ratio of the two subpopulations, say R, can be obtained:

$$f(T) \propto R(T^*) = \frac{P_{SPM}(T^*)}{P_b(T^*)} = -\frac{\int_{(T^*)}^{(T_{final})} f(T)dT}{\int_{(T_0)}^{(T^*)} f(T)dT} \quad \text{(S10)}$$

where T_0 and T_{final} are the extremes of the temperature range covered during the measurement, and the average T_b for the system is the temperature at which $R(T_b)$=1.

References

(1) Langford, J. I.; Wilson, A. J. C. Scherrer after Sixty Years: A Survey and Some New Results in the Determination of Crystallite Size. *J Appl Crystallogr* **2016**, *11*, 1536–1545. https://doi.org/10.1061/9780784479896.140.

(2) Muscas, G.; Singh, G.; Glomm, W. R.; Mathieu, R.; Kumar, P. A.; Concas, G.; Agostinelli, E.; Peddis, D. Tuning the Size and Shape of Oxide Nanoparticles by Controlling Oxygen Content in the Reaction Environment: Morphological Analysis by Aspect Maps. *Chemistry of Materials* **2015**, *27* (6), 1982–1990. https://doi.org/10.1021/cm5038815.

(3) René de Cotret, L. P.; Siwick, B. J. A General Method for Baseline-Removal in Ultrafast Electron Powder Diffraction Data Using the Dual-Tree Complex Wavelet Transform. *Structural Dynamics* **2017**, *4* (4). https://doi.org/10.1063/1.4972518.

(4) René de Cotret, L. P.; Otto, M. R.; Stern, M. J.; Siwick, B. J. An Open-Source Software Ecosystem for the Interactive Exploration of Ultrafast Electron Scattering Data. *Adv Struct Chem Imaging* **2018**, *4* (1), 11. https://doi.org/10.1186/s40679-018-0060-y.

(5) Della Torre, E. *Magnetic Hysteresis*; IEEE, 2000. https://doi.org/10.1109/9780470545195.

(6) Batlle, X.; García del Muro, M.; Tejada, J.; Pfeiffer, H.; Görnert, P.; Sinn, E. Magnetic Study of *M* -Type Doped Barium Ferrite Nanocrystalline Powders. *J Appl Phys* **1993**, *74* (5), 3333–3340. https://doi.org/10.1063/1.354558.

(7) Wohlfarth, E. P. Relations between Different Modes of Acquisition of the Remanent Magnetization of Ferromagnetic Particles. *J Appl Phys* **1958**, *29* (3), 595–596. https://doi.org/10.1063/1.1723232.

(8) García-Otero, J.; Porto, M.; Rivas, J. Henkel Plots of Single-Domain Ferromagnetic Particles. *J Appl Phys* **2000**, *87* (10), 7376–7381. https://doi.org/10.1063/1.372996.

(9) Concas, G.; Congiu, F.; Muscas, G.; Peddis, D. Determination of Blocking Temperature in Magnetization and Mössbauer Time Scale: A Functional Form Approach. *The Journal of Physical Chemistry C* **2017**, *121* (30), 16541–16548. https://doi.org/10.1021/acs.jpcc.7b01748.
